# HIV Modes of Transmission in Sudan in 2014

**DOI:** 10.15171/ijhpm.2019.91

**Published:** 2019-11-03

**Authors:** Maryam Nasirian, Sina Kianersi, Mohammad Karamouzian, Mohammed Sidahmed, Mohammad Reza Baneshi, Ali Akbar Haghdoost, Hamid Sharifi

**Affiliations:** ^1^Epidemiology and Biostatistics Department, Health School, Isfahan University of Medical Sciences, Isfahan, Iran.; ^2^Infectious Diseases and Tropical Medicine Research Center, Isfahan University of Medical Sciences, Isfahan, Iran.; ^3^Department of Epidemiology and Biostatistics, Indiana University School of Public Health-Bloomington, Bloomington, IN, USA.; ^4^School of Population and Public Health, Faculty of Medicine, University of British Columbia, Vancouver, BC, Canada.; ^5^World Health Organization, Sudan Office, Khartoum, Sudan.; ^6^Modeling in Health Research Center, Institute for Futures Studies in Health, Kerman University of Medical Sciences, Kerman, Iran.; ^7^HIV/STI Surveillance Research Center, and WHO Collaborating Center for HIV Surveillance, Institute for Futures Studies in Health, Kerman University of Medical Sciences, Kerman, Iran.

**Keywords:** Sudan, Modes of Transmission (MoT), HIV

## Abstract

**Background:** In Sudan, where studies on HIV dynamics are few, model projections provide an additional source of information for policy-makers to identify data collection priorities and develop prevention programs. In this study, we aimed to estimate the distribution of new HIV infections by mode of exposure and to identify populations who are disproportionately contributing to the total number of new infections in Sudan.

**Methods:** We applied the modes of transmission (MoT) mathematical model in Sudan to estimate the distribution of new HIV infections among the 15-49 age group for 2014, based on the main routes of exposure to HIV. Data for the MoT model were collected through a systematic review of peer-reviewed articles, grey literature, interviews with key participants and focus groups. We used the MoT uncertainty module to represent uncertainty in model projections and created one general model for the whole nation and 5 sub-models for each region (Northern, Central, Eastern, Kurdufan, and Khartoum regions). We also examined how different service coverages could change HIV incidence rates and distributions in Sudan.

**Results:** The model estimated that about 6000 new HIV infections occurred in Sudan in 2014 (95% CI: 4651-7432). Men who had sex with men (MSM) (30.52%), female sex workers (FSW) (16.37%), and FSW’s clients accounted (19.43%) for most of the new HIV cases. FSW accounted for the highest incidence rate in the Central, Kurdufan, and Khartoum regions; and FSW’s clients had the highest incidence rate in the Eastern and Northern regions. The annual incidence rate of HIV in the total adult population was estimated at 330 per 1 000 000 populations. The incidence rate was at its highest in the Eastern region (980 annual infections per 1 000 000 populations).

**Conclusion:** Although the national HIV incidence rate estimate was relatively low compared to that observed in some sub-Saharan African countries with generalized epidemics, a more severe epidemic existed within certain regions and key populations. HIV burden was mostly concentrated among MSM, FSW, and FSW’s clients both nationally and regionally. Thus, the authorities should pay more attention to key populations and Eastern and Northern regions when developing prevention programs. The findings of this study can improve HIV prevention programs in Sudan.

## Background

The number of people living with HIV (PLHIV) in the Middle East and North Africa (MENA) region is rapidly growing.^1,2^ In 2015, the number of PLHIV was around 36.7 million globally, and there were 2.1 million new HIV infections.^3^ In the same year, the number of PLHIV was 230 000 for the MENA region with 21 000 incident cases.^3^ In MENA, Sudan made up for around 21% of PLHIV in 2013^4^, and there were 5000 new HIV infections in 2014 (0.24 HIV incidence per 1000 people).^5^ In 2016, the same number of new HIV infections was reported with a lower incidence rate.^5^ The most affected Sudanese population was the 15-49 age group.^6^

Our understanding of HIV dynamics in Sudan is limited. However, the recent biobehavioral surveillance surveys and estimation studies among key populations and pregnant women have increased our understanding of the epidemic. In some MENA countries (eg, Iran, Pakistan, and Afghanistan), the HIV epidemic is mainly concentrated among people who inject drugs (PWID),^2^ while in others, such as Morocco, the epidemic is spread mostly via unprotected heterosexual sex.^6^ For Sudan, the consensus opinion of national HIV experts presumed that the epidemic is driven by hetero- and homosexual sex and that PWID play a secondary role in the dynamics of the epidemic.^7^ In 2000, HIV prevalence was less than 0.5% in the general adult Sudanese population.^8^ However, it was notably higher in female sex workers (FSW),^9^ men who had sex with men (MSM),^10^ and tuberculosis patients in Eastern regions and Khartoum state.^11^ National statistics in 2010-2011 reported an HIV prevalence of 1.6% and 2.4% among FSW and MSM, respectively.^12^

The dynamics of the HIV epidemic in Sudan are subject to change mostly as a result of the post secession in 2011 sociopolitical changes such as increase in the number of states from 15 to 18 and massive population relocations within and outside Sudan. In addition, lack of HIV knowledge among the key and general populations could deteriorate the state of the epidemic. Integrated Bio-Behavioral Surveys in 2010-2011 in Sudan showed that HIV knowledge was low (3%-40%) among MSM and FSW and also 2010 Sudan Health and Household Survey (SHHS) showed it was very low (6.7%) among the general population.^13,14^ To identify the priorities for developing preventive intervention programs, it is imperative to identify the prime key populations at risk of HIV and to monitor the changes in modes of HIV transmission in the context of Sudan. Therefore, we aimed to contribute to the existing evidence through the application of the modes of transmission (MoT) model,^15^ which identifies the key populations with the highest HIV burden, the major routes of HIV acquisition, and new incident cases. The MoT model recommended by the Joint United Nations Programme on HIV/AIDS (UNAIDS) is a mathematical model that uses a wide range of data, including but not limited to population size estimations, HIV prevalence, and sexual and injecting behaviors, to calculate the expected incidence of new HIV infection in the short-term.^16^ The findings of this study can help develop and implement more efficient HIV prevention programs in Sudan.

## Methods

In 2013, we used the MoT to predict the distribution of new HIV infections among the 15-49 age group in Sudan in 2014, based on the main routes of exposure to HIV for 2014 (Table 1).^17^ The MoT analysis was done nationally and then for 5 (Central, Eastern, Khartoum, Kordufan, Northern) subnational regions of Sudan.

**Table 1:** Definitions of the Main HIV Exposure Groups

**Groups**	**Definition**
PWID	Adults (men and women) who injected drugs at least once in the past year and for whom the most important means of HIV acquisition was needle sharing
Partners of PWID	Regular sex partners of those who inject drugs
FSW	Adult women who have exchanged sex at least once in the last year for money, food, accommodation, or anything else
Clients of FSW	Adult men who have paid for sex with a sex worker in the last 12 months
Partners of FSW’s clients	Regular, non-commercial, sex partners of clients of sex workers
MSM	Adult men who have had sex with another man at least once in the last year
Female partners of MSM	Regular female sex partners of those MSM who also have sex with women
CHS	Adults (men and women) who have had more than one non-commercial (casual/multiple) sexual partner in the last year.
Partners of CHS	Regular sex partners of people who engage in CHS
Stable heterosexual couples	Adults who are currently in stable heterosexual relationships, without commercial or other casual partners, ie, adults with current low-risk behavior (including those with former high-risk behavior)
No risk	Adults who have been at no risk of acquiring HIV in the last year, ie, those who do not inject drugs and are not currently involved in any sexual activity
Medical injections	Adults who have received at least one medical injection in the last 12 months. In the absence of data it can be assumed to include the total adult population
Blood transfusion	Adults who received a blood transfusion in the last 12 months

Abbreviations: PWID, people who inject drugs; FSW, female sex workers; MSM, men who have sex with men; CHS, casual heterosexual sex.

### The Modes of Transmission Model Structure and Parameters

To process the analysis, the MoT model requires 7 inputs for 13 exposure groups (Table 1) and total 15-49 year-old population: (*i* ) population size; (*ii* ) STIs prevalence; (*iii* ) HIV prevalence; (*iv* ) parameters of risky behaviors, including average annual number of partners, number of high-risk behaviors (eg, sexual or injecting contacts) per partner as well as proportion of sexual or injecting acts that were protected; (*v* ) transmission probability of HIV based on the exposure path (eg, unsafe injection or sex, medical injection or blood transfusion); (*vi* ) proportion circumcised; and (*vii* ) antiretroviral therapy (ART) coverage.

### Modes of Transmission Data Sources in Sudan

Sudan’s epidemiological data for the MoT model were collected through a systematic review of peer-reviewed and grey literature as well as expert opinion through interviews and focus groups discussions with key participants. Participants were 6 individuals from the Ministry of Health, 3 from Sudan World Health Organization (WHO), and 2 from Sudan UNAIDS. They were expert in the field of HIV in Sudan and had at least 5 years of experience in this filed.

1. Population size estimations: With reference to Sudan’s Central Bureau of Statistics, the population of Sudan was 36 163 778 in 2013. The 2008 census indicated that almost half of the population were 15-49 years old, of whom 51% were women.^18^ As we did not have updated data on the 15-49 age group in the 5 main regions of Sudan, for both the original and the regional models, we assumed that the share of each age group in the study was similar to the share of age groups in the total population in 2008.
2. Prevalence of HIV and other sexually transmitted infections (STIs): HIV prevalence in the 15-49 year-old general population, FSW, and MSM was estimated based on the obtained data in antenatal clinics, Sudan household health survey (SHHS; 2010), and integrated biobehavioral surveillance survey (IBBSS; 2011-2012).^9,14^
3. FSW and their clients: Inputs about FSW’s and their clients’ population size, HIV/STI prevalence, number of partners per year, number of acts of exposure per partner per year, percentage of protected acts, and number of people receiving ART were obtained from the national IBBSS study in 2010.^9^ For example, the number of Sudanese FSW’s commercial sex partners range from 2 to 5 in a week. We used self-reported abnormal vaginal discharge prevalence in IBBSS as a proxy indicator for STIs prevalence (an HIV transmission cofactor) in FSW. A systematic review reported that in the MENA region, on average, each FSW has one or less client per day, and a low range of condom use.^19^ A biobehavioral survey in 14 capital cities of Sudan reported a low percent of consistent condom use with clients among FSW in 2012.^9,20^ While more than half of FSW had more than 2 clients per day in Khartoum, an average of 4 clients per week was reported in the Red Sea State. Based on this finding, we assumed each FSW to have around 100 clients per year and 6 sexual acts with each client per year. There was a lack of data on FSW’s clients. Since most of their clients are truck drivers, we assigned this group of customers as a proxy population for parameters of population size and HIV prevalence.^9^ However, data on some characteristics of FSW’s clients and their partners (eg, annual number of clients, number of partners, acts of exposure per partner, and STIs prevalence) were input based on the expert-informant panel discussions.
4. PWID and their partners: Experts in the field of HIV in Sudan assert that PWID are not the key player in HIV transmission. As there was no existing primary data on this population in Sudan, data on PWID were obtained from the experts’ discussion on the international estimations and reviews. In subnational models, the proportion of the population who were PWID was assumed to be equal in each zone.
5. MSM and their female partners: Similar to FSW and their clients, we used the 2010 IBBSS to obtain model inputs on MSM and their female partners. Based on the 2010 IBBSS among MSM, 76.3% of MSM had sex with a woman during the past 6 months. During experts’ discussions this number was used to calculate the number of regular female partners of MSM (19.1% in the national model). While in the regional model, this percentage was estimated lower Supplementary file 1, Table S1).
6. Casual heterosexual sex (CHS): We selected university students as a proxy population for CHS and their partners for all the used parameters in MoT, except for population size.^21^ Additionally, parameters were adjusted based on experts’ opinions. In the expert panel, we asked experts to modify the parameters as university students were younger and also maybe the behaviors of them were different with CHS.
7. Circumcision rate: Sudanese people are predominantly Muslim. Hence, we assumed male circumcision practice to be 100%.
8. Number of people on ART: The proportion of PLHIV receiving ART was acquired from Sudan national AIDS program (Synonymous Non-synonymous Analysis Program, SNAP 2012). However, ART coverage for FSW and MSM was obtained from the 2010 IBBSS. We assumed the same ART coverage across groups; however, it was different between regions.
9. HIV transmission probability per act of exposure: Probability of sexual transmission from men to women and women to men was 0.001 and 0.0004, respectively. The transmission probability among PWID and MSM was the same as 0.01. The transmission probability was 4 times higher in the presence of STIs, while it was reduced by 60% with circumcision. Also, ART reduces transmission probability through heterosexual, homosexual, and needles as 96%, 90%, 80%, respectively.^17^ The same estimates were applied in the subnational models.

Furthermore, experts’ opinions helped us make estimates on stable heterosexual couples, medical injections, blood transfusion, and no-risk subpopulation. (see Supplementary file 1, Table S1 for further information about inputs of each population).

### Data Availability and Quality Scores

Data were quality-checked by UNAIDS Epi review tool and were stored in an Excel sheet.^22^ Data availability and quality score was 55% and 1.6/3, respectively, which are relatively low (Supplementary file 1; Table S2 and Figure S1).

### Modes of Transmission Model Assumptions

In the MoT model, the risk of infection in a susceptible individual is assumed as a simple binomial function of the number of partners and number of sex acts per partner. Therefore, the expected number of new HIV infections in the coming year is estimated using the following equation:

I = S[1-{B(1-Beta’) ^a(1-v) +(1-B) (1-Beta) ^a(1-v) + (1-P)} ^n]

where I is the incidence of HIV in the target population, S is the number susceptible individuals in the target population, P is HIV prevalence in the partner population, B is STIs prevalence in the target or partner population, β’ and β are HIV transmission probability during a single contact in the presence or absence of an STI respectively (β’ = β if transmission route is needle-sharing), υ is protected act proportion by condom or sterile needles, a is the number of contacts per partner, and n is the number of partners.

### Uncertainty Analysis

To explore the uncertainty associated with the input parameters on the reliability of the MoT model outputs, we used the uncertainty module in which the determined MoT inputs are allowed to vary simultaneously and randomly within a range of uncertainty for several runs (typically 500-1000 runs). The estimated number of new HIV infections and total incidence rate was different in each run. We used the range of the total number of new HIV infections among adults (aged 15-49 years) from the Spectrum model, which was used in 2008 to generate Sudan’s official national estimates of the HIV epidemic.^23^ The model runs that fit within this range were selected, median was calculated, and the uncertainty level was described by plausibility bounds (2.5 and 97.5 percentiles).

### Application of the Modes of Transmission at Subnational Level

With respect to the difference between exposure groups and their related variables in various regions (eg, Northern, Central, Eastern, Kurdufan, and Khartoum) in Sudan, we created one general model for the whole nation and 5 sub-models for each region. Then, we compared the results of the subnational models with each other and with those of the national model.

### Intervention Scenarios

We also illustrated potential benefits of increased service coverage in 2014 on HIV occurrence in Sudan in that same year. We quantified the extent to which increasing the proportion of protected acts (ie, safe injections and condom use) among the most affected groups in Sudan (eg, PWID, FSW and their clients, MSM, and stable and CHS) could have reduced HIV incidence across the country.

### Validation of Model Outputs

We compared MoT outputs with Spectrum modeling results (2013) and led expert group discussions.

## Results

The MoT model estimated that 6012 new HIV infections would occur in Sudan in 2014 (95% CI: 4651-7432). Based on the model, FSW, their clients and partners of FSW’s clients accounted for 16.05%, 21.65% and 9.27% of the new HIV infections, respectively. MSM and their female partners accounted for 30.51% and 0.95% of the new HIV infections, respectively. PWID and their partners, however, only took up 0.43% and 0.04% of the new infections in that turn. Furthermore, 10.99% and 4.95% of HIV incidence cases were attributed to those having CHS and their partners, respectively while stable heterosexual couples accounted for 7.84% of new HIV infections. The model also estimated that around 912 cases (~15.2%) of the new HIV infections would occur among the stable sexual partners of PWID, clients of FSW, MSM, and the CHS group; corresponding to rate of 480 per 1 000 000. Despite contribution of female partner of MSM, PWID and their partners to total incidence being low, the risk of infection among individuals in those particular groups is high. It was estimated that no new case of HIV infection would occur among blood recipients, while those taking medical injections accounted for 0.02% of the new infections in the model’s projection (Table 2).

**Table 2:** Expected Number, Percentage, and Incidence Rate of New HIV Infections Per 1 000 000 by Exposure Groups in National Model in 2014

**Exposure Group**	**Population Size**	**Number of New HIV Infections**	**Share in National New HIV Infections (as % With 95% CI)**	**Incidence Rate Per 1** **000** **000**
PWID	986	27	0.43 (0.15-1.08)	27 770
Partners of PWID	484	1	0.04 (0.02-0.07)	2070
FSW	212 462	984	16.05 (10.90-21.49)	4630
Clients of FSW	1 487 235	1168	21.65 (11.38-24.11)	790
Partners of FSW’s clients	817 979	567	9.27 (6.28-13.06)	690
MSM	131 998	1835	30.51 (21.78-43.67)	13 900
Female partners of MSM	25 212	43	0.95 (0.67-1.33)	1700
CHS	2 773 762	669	10.99 (7.14-16.28)	240
Partners of CHS	1 070 448	301	4.95 (3.52-6.85)	280
Stable heterosexual couples	5 081 011	410	7.84 (4.05-13.68)	80
No risk	6 480 312	0	0.00	0
Medical injections	18 081 889	6	0.02 (0.017-0.03)	0
Blood transfusions	291 920	0	0.00	0
Total adult population	18 081 889	6012 (4651-7432)	100	330

Abbreviations: PWID, people who inject drugs; FSW, female sex workers; MSM, men who have sex with men; CHS, casual heterosexual sex.

**Figure 1:**
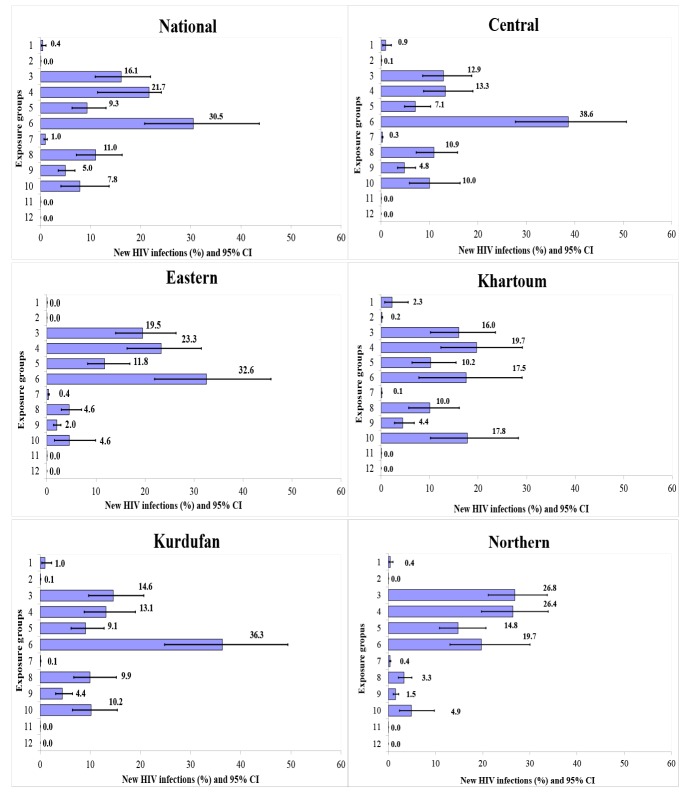
The Estimated Share in National New HIV Infections, by Exposure Group and Sub-national Region, For 2014. Note: lines indicate the 95% CIs on the best estimates represented by bars. Abbreviations: PWID, people who inject drugs; FSW, female sex workers; MSM, men who have sex with men; CHS, casual heterosexual sex.

When applying the model to each sub-region, the percentage of new HIV infections among people who had stable heterosexual relationship in Khartoum, Central, and Kordufan regions was higher than the national estimation respectively. FSW and their clients in Northern and Eastern regions had high rates of new HIV infections in comparison with national estimation. The percentage of new HIV infection among MSM in Central, Kordufan, and Eastern regions as well as that of PWID in Khartoum was higher than the national estimation. Conversely, the lowest percentage of new HIV infections among FSW and MSM was in central and Khartoum. Northern and Eastern had the least percentage of new HIV infection among CHS and stable heterosexual couples, respectively (Figure 1) (Supplementary file 1; Table S3 and Figure S2).

**Figure 2:**
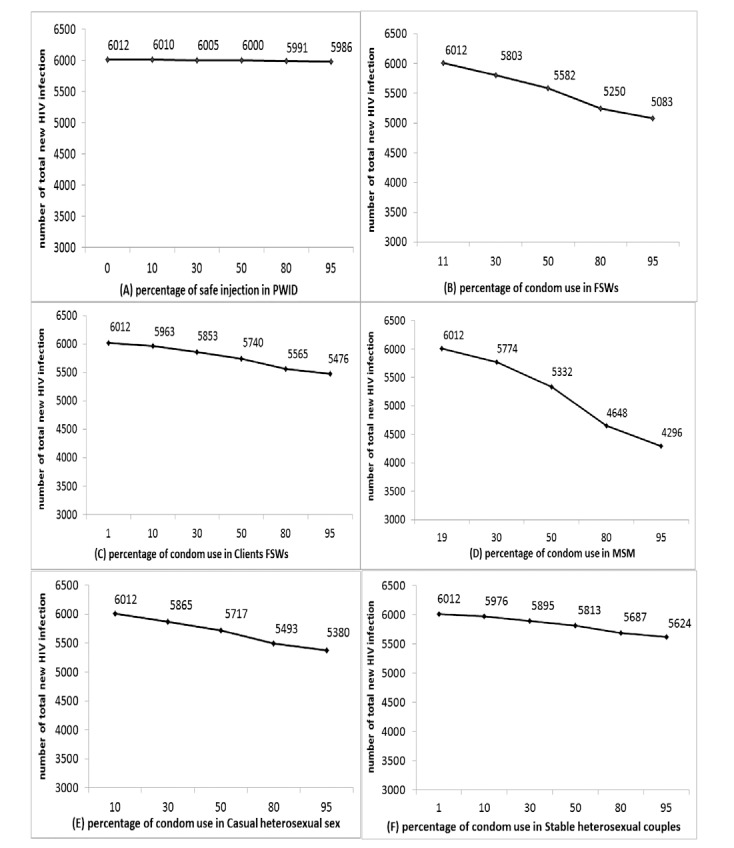
Annual Incidence Rate of HIV Infection Per 1 000 000 and Number of New HIV Cases for 2014, at the National and Sub-national Levels.

The incidence rate of HIV in the total adult population (15-49 age-group) was estimated at 330 per 1 000 000 person-year, while this rate was higher in Eastern and Northern regions (Figure 2).

**Figure 3:**
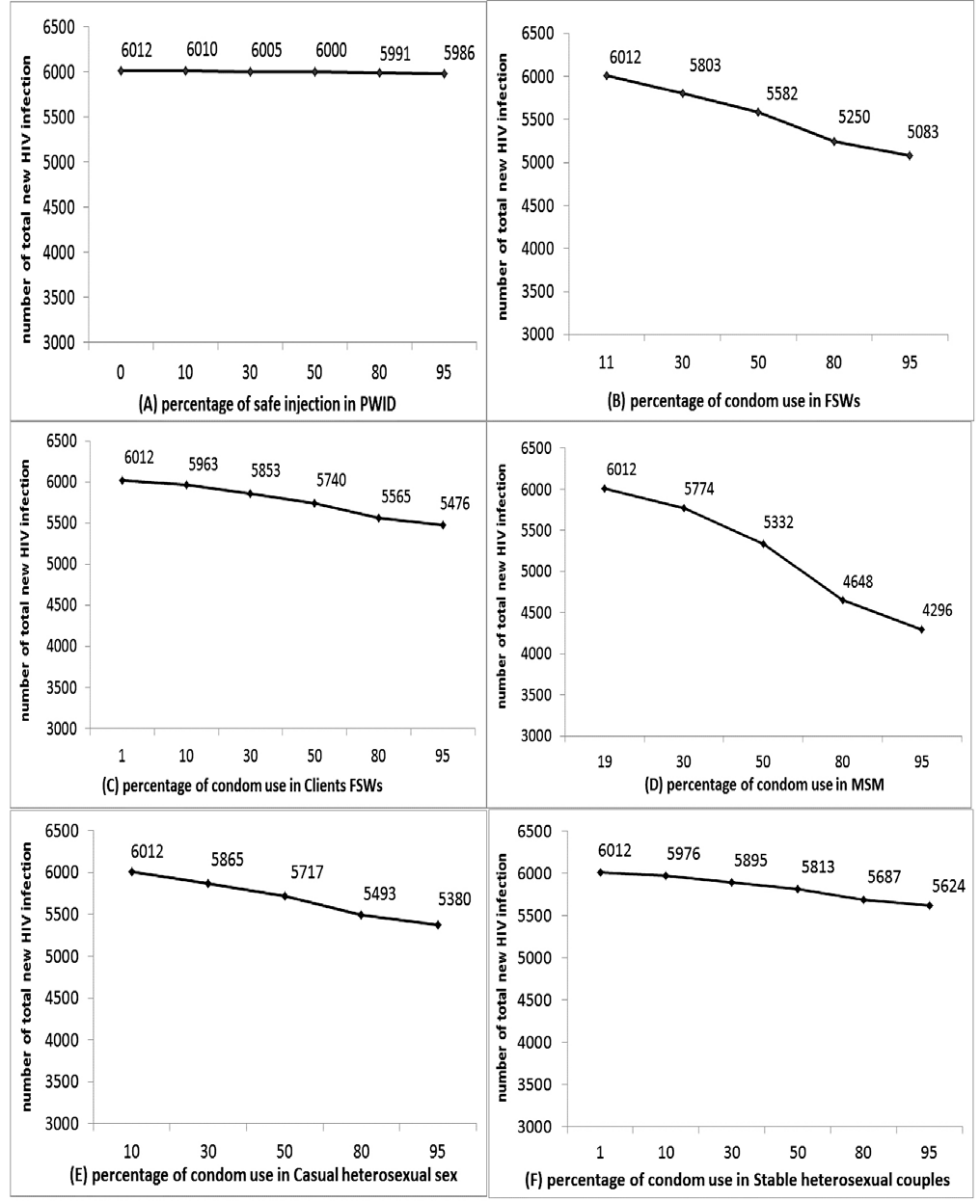
Potential Impact of Increasing Condom Use Coverage on the Number of New HIV Infections Disaggregated by Exposure Groups.

We also examined the hypothetical impact of changes in services of condom and sterile syringes distribution on HIV occurrence among key populations in that same year (2014), at the national level. If the injection safety program coverage rose from 0% to 95% among PWID, the number of HIV infections would have decreased by 93% and 100% among PWID and their sexual partners, respectively. However, this change in coverage had very little impact on the total number of new HIV infection in Sudan (by 0.43%). The MoT also estimated that increasing condom use from 11% to 95% among FSW would have prevented 15.5% fewer new HIV infections in 2014 approximately. In addition, if among clients of FSW, condom use with their stable partner had been 95% instead of the estimated 10.8%, there would have been 8.9% fewer new HIV infections. Our findings also demonstrated that if condom use coverage had risen from 19% to 95% among MSM, approximately 28.5% of new HIV infections would be averted in Sudan. Had condom use coverage been 95% instead of the estimated 10% among CHS, then the number of new HIV infections would have been 10.5% lower than our best estimate. Finally, had condom use coverage among stable heterosexual couples been 95% instead of 1%, there would have been 6.5% fewer new HIV infections in 2014 (Figure 3).

## Discussion

We used the MoT model to provide a better picture of the HIV epidemic spread and its main modes of exposure in Sudan. Over 6000 new infections were estimated to have occurred in 2014, of which nearly a third were among MSM, and more than a third among FSW and their clients. However, there were variations between sub-regions, with a higher contribution of FSW and clients in the northern and eastern regions.

Our findings are comparable with reported estimates. UNAIDS, using the Spectrum Aids Incidence Model and Estimation and Projection Package, estimated 5196 (2328-9138) new HIV infections in 2013.^24^ Moreover, among adults, the number of new HIV infections was estimated at 4400 (1200-9500) and cumulative incidence per 1 000 000 population was 200 (60-490) in 2016.^5^ The global burden of disease study, also using Estimation and Projection Package estimated 4310 (1250 to 8660) new infections to have occurred in 2015.^25^

In our study, MSM and FSW’s clients had the largest shares in the national number of new HIV infections, both nation-wide and in subnational regions. The model estimated zero new infection associated with blood transfusion and medical injection exposure groups.

Among Sudan’s subnational regions, the incidence rate was at its highest in the Eastern region. At the regional level, FSW accounted for the highest incidence rate in the Central, Kurdufan, and Khartoum regions; and FSW’s clients had the highest incidence rate in the Eastern and Northern regions. These similarities between regions are consistent with IBBSS (2011-2012) results. The HIV prevalence for FSW in Kurdufan was between 1% and 1.5%, while it was between 0.2% and 1% in the Central region. In addition, HIV prevalence among FSW in one Eastern site was estimated at 0.5%, while it ranged from zero to 0.7 % in Northern region.^9^ Even though sex work is usually more frequent in larger cities, we found the lowest value of HIV incidence rate in Khartoum.^20^ Data suggest that condom use decreased in Khartoum between 2008 and 2011.^9^ These contradictions call for further investigations. Regions with higher prevalence share borders with countries where the HIV incidence and prevalence are greater. It is possible that more FSW from Ethiopia enter the country through the Eastern region, which could potentially explain the higher HIV incidence rate in FSW clients in this region. The average number of one or less client per day for FSW may mask a small number of sex workers who have even more unprotected client contacts and correspondingly play an important role in HIV transmission. Moreover, HIV counseling and testing has been reported to be poor for Sudanese FSW.^20^ Hence, FSW are highly exposed to the risk of acquiring HIV infection. For example, untreated STIs in FSW contribute as an important HIV transmission cofactor in ongoing HIV spread, while the percent of FSW seeking care for STI symptoms fluctuated between 20%-100% in different cities of Sudan.^9^

Despite the MSM population being small in relation to that of clients or of FSW, their contribution to the epidemic is very high, due to the very high transmission probability and low condom use in this population. Even more so than FSW, MSM had a large share of new HIV infections in Sudan in 2014. Similar to other MENA countries, such as Morocco, Egypt, Tunisia, Kenya, Pakistan, and Yemen,^1,2,26^ in Sudan, HIV prevalence was also estimated to have increased among MSM between 2003 and 2014.^27^ In 2009, in Khartoum, HIV prevalence was 2.3% in MSM.^28^ As a recent cross-sectional study found that condom use among Sudanese men is low,^29^ new surveys evaluated the evolution of the HIV epidemic among Sudanese MSM. This larger share of new HIV infections in MSM, compared to FSW, might be due to the increment of condom use among FSW and the decrement of this preventive behavior in MSM.^5,9,19^ The trends for HIV prevalence in these groups support this hypothesis.^5,19^

Our model indicated a high incidence rate for Sudanese PWID, although this contributed only around 29 new HIV infections, due to the small size of PWID population. However, the quality and representativeness of input data on HIV prevalence and population size of Sudanese PWID was poor, leaving substantial uncertainty in the share of this group in the presented MoT results.^30^ Although PWID, their partners, and medical injection had the lowest number of HIV infection both nationally and regionally, the dynamics of HIV infection did not follow the same pattern throughout the nation.

We acknowledge important limitations of our study. As always, reliability of a MoT analysis depends critically on the quality, representativeness, and generalizability of the input data, which in the current analysis for Sudan in 2014 were relatively uncertain and probably poor (despite being complemented by experts’ opinion). For instance, vaginal discharge prevalence which used as a proxy indicator for STIs prevalence in FSW is not specific to STIs in women. Second, ART data for MSM and FSW came from IBBSS, which was performed among accessible key populations, in whom ART coverage may have been higher than among all FSW and MSM. However, we thrived to find the best available evidence in Sudan and to address the lack of data for some estimates, we ran several focus group discussions with HIV experts in the country. Although we initiated our study in 2013, it took time to make connections and obtain permissions to use and publish the data from relevant partners and experts (Sudan’s UNAIDS, Sudan’s WHO office, Sudan’s Ministry of Health, and Sudan’s national HIV office).

Some other limitations are inherent to the MoT model.^16^ The model assumes that the infection risk is uniform within each group and individuals are only at risk from a single source, thus ignoring patterns of mixing and gradients in risk behaviours by demographic, social and economic variables. Furthermore, the MoT assumes that infectiousness is constant across stages of HIV infection. Since the model estimates are for one year and do not include the secondary infections generated from those new cases in this and the next year,^16^ its results cannot detect the true drivers of the epidemic over its history or guide prioritization of HIV prevention efforts into the future.^31^ Finally, the MoT tool was not designed to investigate the impact of HIV prevention interventions, and our attempt to do so in the form of counterfactual scenarios of changed condom usage rates or changed coverage of sterile syringes is at best a rough approximation of the impact expected from interventions achieving these, through long-term HIV epidemic dynamics.

Indeed, HIV Data validity is an important barrier to HIV prevention, care, treatment efforts in the MENA region. However, we believe it is important to recognize the context of Sudan that is dealing with internal conflicts when it comes to HIV data. In such settings with numerous political, cultural, religious, and other contextual complexities surrounding HIV, as well as with limited resources, running reliable original studies among marginalized populations is difficult. Despite these limitations, we believe we have presented the best possible estimates given available data, thus providing valuable insight into the contribution of various risk factors, population groups and regions in Sudan’s national epidemic as of 2014. To refine and update this MoT analysis, collecting detailed and accurate epidemiological and program data is an urgent priority.

## Conclusion

In countries where studies on HIV dynamics are few, estimates from the MoT model are a useful source of information for policy-makers, even when there are limitations in national epidemiological and programmatic data. The application of the MoT model in Sudan provides estimates of the burden of the HIV epidemic among different populations. The overall HIV incidence at the national level in 2014 was below the threshold of a generalized epidemics of sub-Saharan Africa (defined as HIV prevalence above >1% in the general population)^32^; however, a more severe epidemic existed in certain regions (the Eastern and Northern regions) and key populations. The burden of the infection was mostly concentrated in the key populations MSM, FSW and clients of FSW, both nationally and regionally. Prevention programs that advocate condom use in these key populations and regions may considerably decrease new HIV infections.

## Ethical issues

This study used statistical modeling of secondary data and therefore, no ethical approval was not required.

## Competing interests

Authors declare that they have no competing interests.

## Authors’ contributions

Design and conduct the survey: MN, MS, MRB, AAH, HSH; Data collection: MS, HS; Data analysis: MN, SK; Supervision: HS, AAH; Writing-original draft: MN, SK, MK, HS; Writing, review, and editing: MN, SK, MK, MS, MRB, AAH, HSH; Grant: AAH, HS. All authors read the manuscript and approved the final version of the manuscript.

## Authors’ affiliations

^1^Epidemiology and Biostatistics Department, Health School, Isfahan University of Medical Sciences, Isfahan, Iran. ^2^Infectious Diseases and Tropical Medicine Research Center, Isfahan University of Medical Sciences, Isfahan, Iran. ^3^Department of Epidemiology and Biostatistics, Indiana University School of Public Health-Bloomington, Bloomington, IN, USA. ^4^School of Population and Public Health, Faculty of Medicine, University of British Columbia, Vancouver, BC, Canada. ^5^World Health Organization, Sudan Office, Khartoum, Sudan. ^6^Modeling in Health Research Center, Institute for Futures Studies in Health, Kerman University of Medical Sciences, Kerman, Iran. ^7^HIV/STI Surveillance Research Center, and WHO Collaborating Center for HIV Surveillance, Institute for Futures Studies in Health, Kerman University of Medical Sciences, Kerman, Iran.

## Supplementary files


Supplementary file 1 contains Tables S1-S3 and Figures S1-S2.

## Key messages

**Implications for policy makers**

- The application of the modes of transmission (MoT) model in Sudan can provide an additional source of information for policy-makers to plan HIV prevention programs, at subnational and national levels. Our MoT results for year 2014 suggested that the Eastern and Northern regions had the highest annual HIV incidence.
- Although to date, female sex workers (FSW) and MSM (men who had sex with men) have been identified as 2 major key populations in Sudan, our results illustrate that the distribution of risk groups differed among regions. For example, in the Northern and Eastern regions, HIV prevention programs should be prioritized to clients of FSW.
- The model indicated that an 80% increase in condom use among FSW and MSM would have substantially decreased the number of new HIV cases in Sudan.

**Implications for the public**

The estimates of the modes of transmission (MoT) model provide information on the HIV infection dynamics in Sudan. They are helpful for planning and evaluating intervention programs. Moreover, they may be useful for researchers in guiding and prioritizing research questions.
