# Supplementary material for: HIV Modes of Transmission in Sudan in 2014
Source: Int J Health Policy Manag. 2019 Nov 3;9(3):108–15. doi: 10.15171/ijhpm.2019.91 (PMC7093043; doi:10.15171/ijhpm.2019.91)
Supplement: Supplementary file 1 — contains Tables S1-S3 and Figures S1-S2. [file ijhpm-9-108-s001.pdf]

Supplementary file 1

Table S1. Input parameters for HIV Modes of Transmission among Sudan and Sub-national Regions

Region	Exposure group	Population size			HIV prevalence			STIs prevalence			Number of partners per year			Number of acts of exposure per partner per year			Percentage of acts that are protected			Number of people receiving ART		
		Value(N)	Uncertainty Ranges(%)	Justification or Reference	Value(%)	Uncertainty Ranges(%)	Justification or Reference	Value(%)	Uncertainty Ranges(%)	Justification or Reference	Value(N)	Uncertainty Ranges(%)	Justification or Reference	Value(N)	Uncertainty Ranges(%)	Justification or Reference	Value(%)	Uncertainty Ranges(%)	Justification or Reference	Value(N)	Uncertainty Ranges(%)	Justification or Reference
National	PWIDs	986	20	EO[1]	6.00	50	EO[1]	10.0	50	EO[1]	2	50	EO[1]	30	50	EO[1]	0.00	50	EO[1]	4	-	SNAP[2]
	PWIDs' Partners	484	20	EO[1]	3.00	50	EO[1]	NA	-	-	1	-	EO[1]	30	10	EO[1]	0.00	20	EO[1]	1	-	SNAP[2]
	FSW	212,462	20	PSE[3] 2012	1.60	20	IBBSS[4] 2010	28.0	20	IBBSS[4] 2010	100	20	WBP[5] 2010; EO[1]	5	10	WBP[5] 2010; EO[1]	11.00	10	IBBSS[4] 2010	189	-	SNAP[2]
	FSW' Clients	1,487,235	20	PSE[3] 2012; EO[1]	0.90	20	IBBST[6] 2008; EO[1]	9.3	20	EO[1]	10	20	EO[1]	8	10	EO[1]	10.80	10	EO[1]	1059	-	SNAP[2]
	Partners of FSW's clients	817,979	20	PSE[2] 2012; EO[1]	0.45	20	IBBST[6] 2008; EO[1]	NA	-	-	1	-	EO[1]	70	10	EO[1]	1.00	5	EO[1]	389	-	SNAP[2]
	MSM	131,998	20	PSE[2] 2012; mapping 2012	2.55	20	IBBSS[4] 2010; Spectrum 2103	15.0	20	IBBSS[4] 2010	10	20	IBBSS[4] 2010	5.2	10	IBBSS[4] 2010	19.00	10	IBBSS[4] 2010	173	-	SNAP[2]
	MSM's Female partners	25,212	20	IBBSS[4] 2010; EO[1]	1.28	20	IBBSS[4] 2010; EO[1]	NA	-	-	1	-	EO[1]	52	10	EO[1]	1.00	5	EO[1]	70	-	SNAP[2]
	(CHS)	2,773,762	20	SCBS[7]; EO[1]	0.50	20	ANC[8] 2010; SHHS[9] 2010; EO[1]	6.0	20	BSS University 2010;EO[1]	2.75	20	EO[1]	30	10	EO[1]	10.00	5	BSS University 2010; EO[1]	712	-	SNAP[2]
	CHS's partners	1,070,448	20	SCBS[7]; EO[1]	0.25	20	ANC[8] 2010; SHHS[9] 2010; EO[1]	NA	-	-	1	-	EO[1]	70	10	EO[1]	1.00	5	EO[1]	133	-	SNAP[2]
	Stable heterosexual couples	5,081,011	20	SCBS[7]; EO[1]	0.15	20	ANC[8] 2010; SHHS[9] 2010;	5.0	20	-	1	-	EO[1]	70	10	EO[1]	1.00	5	EO[1]	461	-	SNAP[2]

	No risk	6,480,312	20	SCBS[7]; EO[1]	0.01	0	ANC[8] 2010; SHHS[9] 2010; EO[1]	2.0	0	EO[1]	0	-	EO[1]	0	0	EO[1]	0.00	-	EO[1]	39	-	SNAP[2]
	Medical injections	18,081,889	-	EO[1]	0.01	-	EO[1]	NA	-	-	1	10	-	1	-	-	95.00	-	EO[1]	-	-	-
	Blood transfusions	291,920	-	EO[1]	0.01	10	EO[1]	NA	-	-	1	-	-	1	-	-	100.00	-	EO[1]	-	-	-
**Sub-national**																						
Central	PWIDs	302	20	EO[1]	6.00	50	EO[1]	0.0	50	EO[1]	2	50	EO[1]	30	50	EO[1]	0.00	50	EO[1]	0	-	SNAP[2]
	PWIDs' Partners	150	20	EO[1]	3.00	50	EO[1]	NA	-	-	1	-	EO[1]	30	10	EO[1]	0.00	20	EO[1]	0	-	SNAP[2]
	FSW	46,112	20	PSE[3] 2012	0.80	20	IBBSS[4] 2010	26.0	20	IBBSS[4] 2010	100	20	WBP[5] 2010; EO[1]	5	10	WBP[5] 2010; EO[1]	8.20	10	IBBSS[4] 2010	16	-	SNAP[2]
	FSW' Clients	322,781	20	PSE[3] 2012; EO[1]	0.45	20	IBBST[6] 2008; EO[1]	9.0	20	EO[1]	10	20	EO[1]	8	10	EO[1]	8.00	10	EO[1]	88	-	SNAP[2]
	Partners of FSW's clients	177,529	20	PSE[2] 2012; EO[1]	0.22	20	IBBST[6] 2008; EO[1]	NA	-	-	1	-	EO[1]	70	10	EO[1]	2.00	5	EO[1]	32	-	SNAP[2]
	MSM	20,836	20	PSE[2] 2012; mapping 2012	2.50	20	IBBSS[4] 2010; Spectrum 2103	15.8	20	IBBSS[4] 2010	10	20	IBBSS[4] 2010	5.2	10	IBBSS[4] 2010	5.80	10	IBBSS[4] 2010	14	-	SNAP[2]
	MSM's Female partners	1,042	20	IBBSS[4] 2010; EO[1]	1.25	20	IBBSS[4] 2010; EO[1]	NA	-	-	1	-	EO[1]	52	10	EO[1]	2.00	5	EO[1]	6	-	SNAP[2]
	(CHS)	482,463	20	SCBS[7]; EO[1]	0.40	20	ANC[8] 2010; SHHS[9] 2010; EO[1]	6.0	20	BSS University 2010;EO[1]	2.75	20	EO[1]	30	10	EO[1]	10.00	5	BSS Univerrsity 2010; EO[1]	59	-	SNAP[2]
	CHS's partners	186,439	20	SCBS[7]; EO[1]	0.26	20	ANC[8] 2010; SHHS[9] 2010; EO[1]	NA	-	-	1	-	EO[1]	70	10	EO[1]	2.00	5	EO[1]	11	-	SNAP[2]
	Stable heterosexual couples	969,367	20	SCBS[7]; EO[1]	0.13	20	ANC[8] 2010; SHHS[9] 2010;	5.0	20	-	1	-	EO[1]	70	10	EO[1]	2.00	5	EO[1]	38	-	SNAP[2]
	No risk	1,208,805	20	SCBS[7]; EO[1]	0.01	0	ANC[8] 2010; SHHS[9] 2010; EO[1]	2.0	0	EO[1]	0	-	EO[1]	0	0	EO[1]	0.00	-	EO[1]	3	-	SNAP[2]
	Medical injections	3,415,669	-	EO[1]	0.01	-	EO[1]	NA	-	-	1	10	-	1	-	-	95.00	-	EO[1]	-	-	-

	Blood transfusions	59,837	-	EO[1]	0.01	10	EO[1]	NA	-	-	1	-	-	1	-	-	100.00	-	EO[1]	-	-	-
**Eastern**	PWIDs	35	20	EO[1]	6.00	50	EO[1]	0.0	50	EO[1]	2	50	EO[1]	30	50	EO[1]	0.00	50	EO[1]	1	-	SNAP[2]
	PWIDs' Partners	16	20	EO[1]	3.00	50	EO[1]	NA	-	-	1	-	EO[1]	30	10	EO[1]	0.00	20	EO[1]	0	-	SNAP[2]
	FSW	31,441	20	PSE[3] 2012	6.20	20	IBBSS[4] 2010	25.0	20	IBBSS[4] 2010	100	20	WBP[5] 2010; EO[1]	5	10	WBP[5] 2010; EO[1]	8.50	10	IBBSS[4] 2010	40	-	SNAP[2]
	FSW' Clients	220,086	20	PSE[3] 2012; EO[1]	3.20	20	IBBST[6] 2008; EO[1]	8.0	20	EO[1]	10	20	EO[1]	8	10	EO[1]	8.50	10	EO[1]	222	-	SNAP[2]
	Partners of FSW's clients	121,047	20	PSE[2] 2012; EO[1]	1.25	20	IBBST[6] 2008; EO[1]	NA	-	-	1	-	EO[1]	70	10	EO[1]	2.00	5	EO[1]	82	-	SNAP[2]
	MSM	30,917	20	PSE[2] 2012; mapping 2012	3.80	20	IBBSS[4] 2010; Spectrum 2103	24.6	20	IBBSS[4] 2010	10	20	IBBSS[4] 2010	5.2	10	IBBSS[4] 2010	15.00	10	IBBSS[4] 2010	36	-	SNAP[2]
	MSM's Female partners	2,164	20	IBBSS[4] 2010; EO[1]	1.80	20	IBBSS[4] 2010; EO[1]	NA	-	-	1	-	EO[1]	52	10	EO[1]	2.00	5	EO[1]	15	-	SNAP[2]
	(CHS)	390,390	20	SCBS[7]; EO[1]	0.60	20	ANC[8] 2010; SHHS[9] 2010; EO[1]	6.0	20	BSS University 2010;EO[1]	2.75	20	EO[1]	30	10	EO[1]	10.00	5	BSS University 2010; EO[1]	149	-	SNAP[2]
	CHS's partners	151,527	20	SCBS[7]; EO[1]	0.30	20	ANC[8] 2010; SHHS[9] 2010; EO[1]	NA	-	-	1	-	EO[1]	70	10	EO[1]	2.00	5	EO[1]	28	-	SNAP[2]
	Stable heterosexual couples	721,435	20	SCBS[7]; EO[1]	0.31	20	ANC[8] 2010; SHHS[9] 2010;	5.0	20	-	1	-	EO[1]	70	10	EO[1]	2.00	5	EO[1]	97	-	SNAP[2]
	No risk	947,023	20	SCBS[7]; EO[1]	0.01	0	ANC[8] 2010; SHHS[9] 2010; EO[1]	2.0	0	EO[1]	0	-	EO[1]	0	0	EO[1]	0.00	-	EO[1]	8	-	SNAP[2]
	Medical injections	2,620,066	-	EO[1]	0.01	-	EO[1]	NA	-	-	1	10	-	1	-	-	95.00	-	EO[1]	-	-	-
	Blood transfusions	28,553	-	EO[1]	0.01	10	EO[1]	NA	-	-	1	-	-	1	-	-	100.00	-	EO[1]	-	-	-
Khartoum	PWIDs	301	20	EO[1]	6.00	50	EO[1]	0.0	50	EO[1]	2	50	EO[1]	30	50	EO[1]	0.00	50	EO[1]	2	-	SNAP[2]
	PWIDs' Partners	149	20	EO[1]	3.00	50	EO[1]	NA	-	-	1	-	EO[1]	30	10	EO[1]	0.00	20	EO[1]	1	-	SNAP[2]

	FSW	37,575	20	PSE[3] 2012	0.95	20	IBBSS[4] 2010	17.0	20	IBBSS[4] 2010	100	20	WBP[5] 2010; EO[1]	5	10	WBP[5] 2010; EO[1]	18.60	10	IBBSS[4] 2010	101	-	SNAP[2]
	FSW' Clients	263,026	20	PSE[3] 2012; EO[1]	0.50	20	IBBST[6] 2008; EO[1]	8.0	20	EO[1]	10	20	EO[1]	8	10	EO[1]	18.00	10	EO[1]	565	-	SNAP[2]
	Partners of FSW's clients	144,664	20	PSE[2] 2012; EO[1]	0.25	20	IBBST[6] 2008; EO[1]	NA	-	-	1	-	EO[1]	70	10	EO[1]	2.00	5	EO[1]	208	-	SNAP[2]
	MSM	28,916	20	PSE[2] 2012; mapping 2012	0.60	20	IBBSS[4] 2010; Spectrum 2103	10.0	20	IBBSS[4] 2010	10	20	IBBSS[4] 2010	5.2	10	IBBSS[4] 2010	6.00	10	IBBSS[4] 2010	92	-	SNAP[2]
	MSM's Female partners	1,533	20	IBBSS[4] 2010; EO[1]	0.30	20	IBBSS[4] 2010; EO[1]	NA	-	-	1	-	EO[1]	52	10	EO[1]	2.00	5	EO[1]	37	-	SNAP[2]
	(CHS)	506,447	20	SCBS[7]; EO[1]	0.20	20	ANC[8] 2010; SHHS[9] 2010; EO[1]	6.0	20	BSS University 2010;EO[1]	2.75	20	EO[1]	30	10	EO[1]	10.00	5	BSS University 2010; EO[1]	380	-	SNAP[2]
	CHS's partners	196,044	20	SCBS[7]; EO[1]	0.18	20	ANC[8] 2010; SHHS[9] 2010; EO[1]	NA	-	-	1	-	EO[1]	70	10	EO[1]	2.00	5	EO[1]	71	-	SNAP[2]
	Stable heterosexual couples	918,466	20	SCBS[7]; EO[1]	0.10	20	ANC[8] 2010; SHHS[9] 2010;	5.0	20	-	1	-	EO[1]	70	10	EO[1]	2.00	5	EO[1]	246	-	SNAP[2]
	No risk	1,166,298	20	SCBS[7]; EO[1]	0.01	0	ANC[8] 2010; SHHS[9] 2010; EO[1]	2.0	0	EO[1]	0	-	EO[1]	0	0	EO[1]	0.00	-	EO[1]	21	-	SNAP[2]
	Medical injections	3,267,398	-	EO[1]	0.01	-	EO[1]	NA	-	-	1	10	-	1	-	-	95.00	-	EO[1]	-	-	-
	Blood transfusions	216,244	-	EO[1]	0.01	10	EO[1]	NA	-	-	1	-	-	1	-	-		-	EO[1]	-	-	-
Kordufan	PWIDs	283	20	EO[1]	6.00	50	EO[1]	0.0	50	EO[1]	2	50	EO[1]	30	50	EO[1]	0.00	50	EO[1]	0	-	SNAP[2]
	PWIDs' Partners	139	20	EO[1]	3.00	50	EO[1]	NA	-	-	1	-	EO[1]	30	10	EO[1]	0.00	20	EO[1]	0	-	SNAP[2]
	FSW	31,376	20	PSE[3] 2012	1.20	20	IBBSS[4] 2010; RDS[10] 2012	25.0	20	IBBSS[4] 2010	100	20	WBP[5] 2010; EO[1]	5	10	WBP[5] 2010; EO[1]	20.00	10	IBBSS[4] 2010	20	-	SNAP[2]
	FSW' Clients	219,630	20	PSE[3] 2012; EO[1]	0.70	20	IBBST[6] 2008; EO[1]	15.0	20	EO[1]	10	20	EO[1]	8	10	EO[1]	18.00	10	EO[1]	115	-	SNAP[2]

	Partners of FSW's clients	120,796	20	PSE[2] 2012; EO[1]	0.35	20	IBBST[6] 2008; EO[1]	NA	-	-	1	-	EO[1]	70	10	EO[1]	2.00	5	EO[1]	42	-	SNAP[2]
	MSM	24,403	20	PSE[2] 2012; mapping 2012	1.90	20	IBBSS[4] 2010; Spectrum 2103	11.0	20	IBBSS[4] 2010	10	20	IBBSS[4] 2010	5.2	10	IBBSS[4] 2010	3.00	10	IBBSS[4] 2010	20	-	SNAP[2]
	MSM's Female partners	488	20	IBBSS[4] 2010; EO[1]	1.00	20	IBBSS[4] 2010; EO[1]	NA	-	-	1	-	EO[1]	52	10	EO[1]	2.00	5	EO[1]	8	-	SNAP[2]
	(CHS)	540,359	20	SCBS[7]; EO[1]	0.30	20	ANC[8] 2010; SHHS[9] 2010; EO[1]	6.0	20	BSS University 2010;EO[1]	2.75	20	EO[1]	30	10	EO[1]	10.00	5	BSS University 2010; EO[1]	77	-	SNAP[2]
	CHS's partners	209,171	20	SCBS[7]; EO[1]	0.20	20	ANC[8] 2010; SHHS[9] 2010; EO[1]	NA	-	-	1	-	EO[1]	70	10	EO[1]	2.00	5	EO[1]	14	-	SNAP[2]
	Stable heterosexual couples	1,074,966	20	SCBS[7]; EO[1]	0.13	20	ANC[8] 2010; SHHS[9] 2010;	5.0	20	-	1	-	EO[1]	70	10	EO[1]	2.00	5	EO[1]	50	-	SNAP[2]
	No risk	1,264,615	20	SCBS[7]; EO[1]	0.01	0	ANC[8] 2010; SHHS[9] 2010; EO[1]	2.0	0	EO[1]	0	-	EO[1]	0	0	EO[1]	0.00	-	EO[1]	4	-	SNAP[2]
	Medical injections	3,486,188	-	EO[1]	0.01	-	EO[1]	NA	-	-	1	10	-	1	-	-	95.00	-	EO[1]	-	-	-
	Blood transfusions	55,775	-	EO[1]	0.01	10	EO[1]	NA	-	-	1	-	-	1	-	-	100.00	-	EO[1]	-	-	-
	PWIDs	103	20	EO[1]	6.00	50	EO[1]	0.0	50	EO[1]	2	50	EO[1]	30	50	EO[1]	0.00	50	EO[1]	0	-	SNAP[2]
	PWIDs' Partners	51	20	EO[1]	3.00	50	EO[1]	NA	-	-	1	-	EO[1]	30	10	EO[1]	0.00	20	EO[1]	0	-	SNAP[2]
	FSW	12,418	20	PSE[3] 2012	5.50	20	IBBSS[4] 2010	27.4	20	IBBSS[4] 2010	100	20	WBP[5] 2010; EO[1]	5	10	WBP[5] 2010; EO[1]	2.70	10	IBBSS[4] 2010	5	-	SNAP[2]
Northern	FSW' Clients	86,929	20	PSE[3] 2012; EO[1]	3.00	20	IBBST[6] 2008; EO[1]	9.0	20	EO[1]	10	20	EO[1]	8	10	EO[1]	16.22	10	EO[1]	30	-	SNAP[2]
	Partners of FSW's clients	47,811	20	PSE[2] 2012; EO[1]	1.50	20	IBBST[6] 2008; EO[1]	NA	-	-	1	-	EO[1]	70	10	EO[1]	5.95	5	EO[1]	11	-	SNAP[2]
	MSM	9,393	20	PSE[2] 2012; mapping 2012	2.50	20	IBBSS[4] 2010; Spectrum 2103	7.0	20	IBBSS[4] 2010	10	20	IBBSS[4] 2010	5.2	10	IBBSS[4] 2010	2.70	10	IBBSS[4] 2010	5	-	SNAP[2]

	MSM's Female partners	1,315	20	IBBSS[4] 2010; EO[1]	1.25	20	IBBSS[4] 2010; EO[1]	NA	-	-	1	-	EO[1]	52	10	EO[1]	1.08	5	EO[1]	2	-	SNAP[2]
	(CHS)	159,423	20	SCBS[7]; EO[1]	0.30	20	ANC[8] 2010; SHHS[9] 2010; EO[1]	6.0	20	BSS University 2010;EO[1]	2.75	20	EO[1]	30	10	EO[1]	10.81	5	BSS University 2010; EO[1]	20	-	SNAP[2]
	CHS's partners	61,084	20	SCBS[7]; EO[1]	0.15	20	ANC[8] 2010; SHHS[9] 2010; EO[1]	NA	-	-	1	-	EO[1]	70	10	EO[1]	2.16	5	EO[1]	4	-	SNAP[2]
	Stable heterosexual couples	301,333	20	SCBS[7]; EO[1]	0.05	20	ANC[8] 2010; SHHS[9] 2010;	5.0	20	-	1	-	EO[1]	70	10	EO[1]	6.49	5	EO[1]	12	-	SNAP[2]
	No risk	377,861	20	SCBS[7]; EO[1]	0.01	0	ANC[8] 2010; SHHS[9] 2010; EO[1]	2.0	0	EO[1]	0	-	EO[1]	0	0	EO[1]	0.54	-	EO[1]	1	-	SNAP[2]
	Medical injections	1,061,407	-	EO[1]	0.01	-	EO[1]	NA	-	-	1	10	-	1	-	-	95.00	-	EO[1]	-	-	-
	Blood transfusions	15,369	-	EO[1]	0.01	10	EO[1]	NA	-	-	1	-	-	1	-	-	100.00	-	EO[1]	-	-	-

[1] EO: Expert Opinion
[2] SNAP: Synonymous Non-synonymous Analysis Program.
[3] PSE: Population Size Estimation; capture re capture method
[4] IBBSS: Integrated Bio-Behavioral Surveillance Survey
[5] WBP: World Bank Publications. Avaliable from: https://openknowledge.worldbank.org/handle/10986/2457 License: CC BY 3.0 IGO."
[6] IBBST: Integrated Bio-Behavioral Survey among Truck Driver
[7] SCBS: Sudan Central Bureau of Statistics
[8] ANC: Antenatal Clinic
[9] SHHS: Sudan House Hold Survey
[10] RDS: Respondent Driven Sampling

Table S2. Data Availability and Quality across all population groups

Population group	Average Score of data quality*
Injecting drug users	1.0
Partners of injecting drug users	1.0
Men having sex with men	2.8
Partners of MSM	1.8
Female sex workers	2.8
Clients of female sex workers	1.5
Partners of clients of female sex workers	1.4
Casual heterosexual sex	2.0
Partners of those engaging in casual sex	1.0
Stable heterosexual sex	1.3
No risk populations	1.3
Medical injections	1.0
Blood Transfusions	1.0
**Average Score of quality of all data**	**1.6**
**Data availability score**	**55%**

* 0=none; 1=poor; 2=limited; 3=good

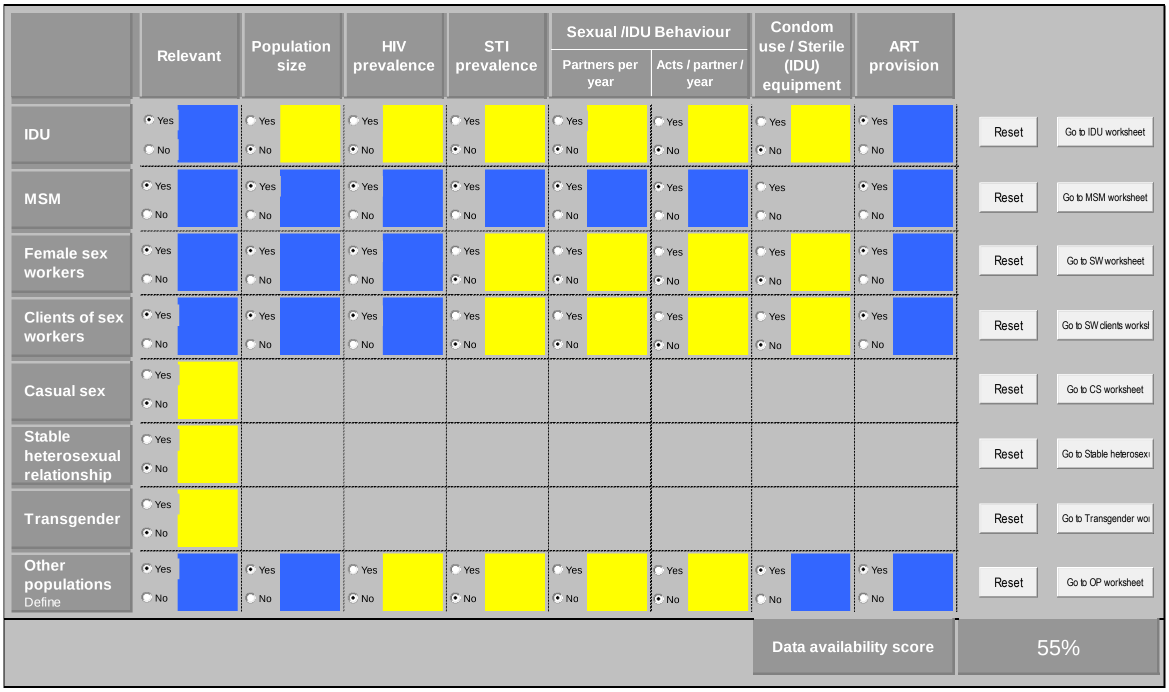

Figure S1. EPi Review Checklist.

Table S3. Expected Number, Percentage, and Incidence rate of New HIV infections per 1000,000 by Exposure Groups in Sub-National models in 2014

Exposure group	Population size	Number of new HIV infections	Share in national new HIV infections (as % with 95% CI)	Incidence rate per 1000,000
**Central**				
PWID	302	9	0.93(0.33-2.09)	2,913
Partners of PWID	150	0.26	0.07(0.03-0.11)	172
FSW	46,112	113	12.88(8.59-18.72)	244
Clients of FSW	322,781	129	13.26(8.77-18.94)	40
Partners of FSW's clients	177,529	62	7.06(4.85-10.21)	35
MSM	20,836	340	38.67(27.74-50.66)	1,632
Female partners of MSM	1,042	2	0.27(0.20-0.37)	171
CHS	482,463	95	10.93(7.25-15.78)	20
partners of CHS	186,439	42	4.81(3.39-7.10)	23
Stable heterosexual couples	969,367	69	9.98(5.80-16.31)	7
No risk	1,208,805	0	0.00	0
Medical injections	3,415,669	1	0.02(0.02-0.03)	0
Blood transfusions	59,837	0	0.00	0
**Total adult population**	**3,415,825**	**861**	**100**	**25**
**Eastern**				
PWID	35	1	0.024(0.005-0.071)	1,894
Partners of PWID	16	0.01	0.001(0.000-0.003)	93
FSW	31,441	513	19.53(14.02-26.43)	1,632
Clients of FSW	220,086	660	23.31(16.36-31.50)	300
Partners of FSW's clients	121,047	298	11.79(8.32-16.92)	246
MSM	30,917	798	32.56(21.98-45.71)	2,580
Female partners of MSM	2,164	7	0.35(0.25-0.49)	301
CHS	390,390	113	4.55(2.95-7.08)	29
partners of CHS	151,527	49	2.04(1.38-2.85)	33
Stable heterosexual couples	721,435	121	4.58(1.55-9.93)	17
No risk	947,023	0	0.00	0
Medical injections	2,620,066	2	0.02(0.01-0.02)	0
Blood transfusions	28,553	0	0.00	0
**Total adult population**	**2,616,080**	**2,562**	**100**	**98**
**Khartoum**				
PWID	301	8	2.26(0.74-5.57)	2,690
Partners of PWID	149	0.23	0.16(0.07-0.28)	154
FSW	37,575	55	16.00(10.17-23.57)	147
Clients of FSW	263,026	72	19.62(12.32-29.13)	27
Partners of FSW's clients	144,664	35	10.20(6.36-15.45)	24
MSM	28,916	56	17.54(7.78-29.07)	194
Female partners of MSM	1,533	0.29	0.12(0.06-0.19)	19
CHS	506,447	33	10.03(5.69-16.10)	7
partners of CHS	196,044	15	4.44(2.76-6.82)	8
Stable heterosexual couples	918,466	39	17.78(10.18-28.30)	4

No risk	1,166,298	0	0.00	0
Medical injections	3,267,398	1	0.04(0.03-0.05)	0
Blood transfusions	216,244	0	0.00	0
**Total adult population**	**3,263,418**	**314**	**100**	**10**
**Kordufan**				
PWID	283	8	0.97(0.35-2.29)	2,913
Partners of PWID	139	0.24	0.07(0.03-0.12)	172
FSW	31,376	116	14.60(9.68-20.67)	370
Clients of FSW	219,630	114	13.13(8.80-18.98)	52
Partners of FSW's clients	120,796	73	9.06(6.18-12.77)	60
MSM	24,403	281	36.33(24.82-49.39)	1,151
Female partners of MSM	488	1	0.10(0.07-0.14)	117
CHS	540,359	79	9.94(6.71-15.19)	15
partners of CHS	209,171	35	4.42(3.09-6.45)	17
Stable heterosexual couples	1,074,966	75	10.16(6.46-15.39)	7
No risk	1,264,615	0	0.00	0
Medical injections	3,486,188	1	0.02(0.02-0.03)	0
Blood transfusions	55,775	0	0.00	0
**Total adult population**	**3,486,227**	**783**	**100**	**22**
**Northern**				
PWID	103	3	0.41(0.14-0.99)	2,913
Partners of PWID	51	0.09	0.03(0.01-0.05)	172
FSW	12,418	212	26.82(21.20-33.73)	1,711
Clients of FSW	86,929	224	26.42(19.74-33.89)	258
Partners of FSW's clients	47,811	110	14.78(10.84-20.69)	231
MSM	9,393	132	19.71(13.07-30.03)	1,406
Female partners of MSM	1,315	2	0.38(0.26-0.52)	144
CHS	159,423	23	3.32(2.14-4.99)	14
partners of CHS	61,084	10	1.53(1.04-2.19)	17
Stable heterosexual couples	301,333	8	4.87(2.36-9.75)	2
No risk	377,861	0	0.00	0
Medical injections	1,061,407	1	0.02(0.02-0.03)	0
Blood transfusions	15,369	0	0.00	0
**Total adult population**	**1,057,723**	**725**	**100**	**69**

Abbreviations: PWID (People who inject drugs); FSW (Female sex workers); MSM (Men who have sex with men); CHS (Casual heterosexual sex)

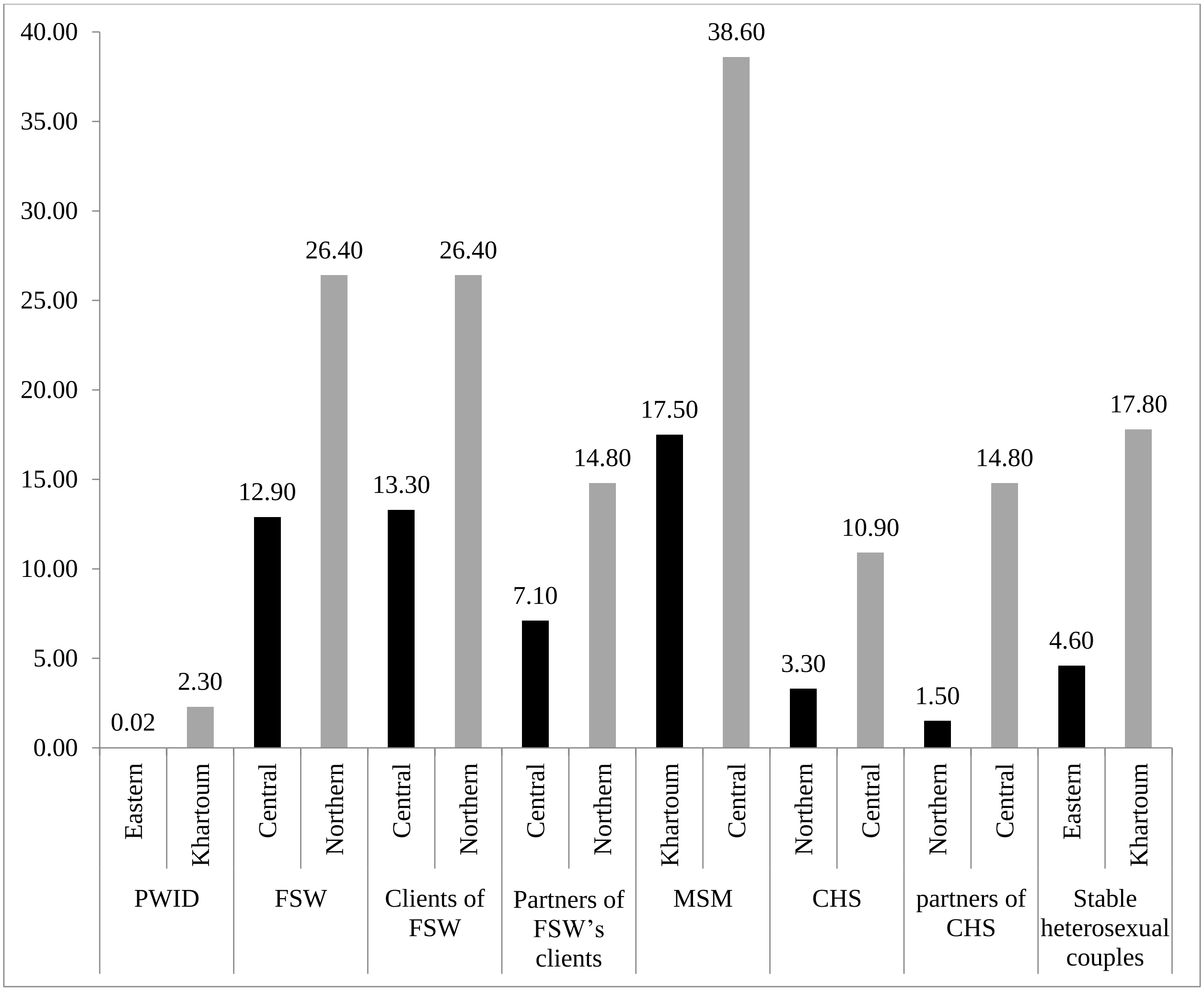

Figure S2. Regions with the highest and lowest percentage of new HIV cases in based on exposure groups.
